# Supplementary material for: Effectiveness of mindfulness-based online therapy or internet-delivered cognitive behavioral therapy compared with treatment as usual among patients with persistent somatic symptoms: Protocol for a randomized controlled trial
Source: PLoS One. 2025 Feb 12;20(2):e0316169. doi: 10.1371/journal.pone.0316169 (PMC11819597; doi:10.1371/journal.pone.0316169)
Supplement: S1 Appendix — (DOCX) [file pone.0316169.s002.docx]

## S2 Appendix:

### Diagnostic criteria for fibromyalgia, chronic fatigue syndrome and post COVID-19 condition

1. **Fibromyalgia: The American College of Rheumatology diagnostic criteria for fibromyalgia**

In this study, we will apply the ICD-10 code M79.7 for fibromyalgia.

Criteria

A patient satisfies diagnostic criteria for fibromyalgia if the following 3 conditions are met:

1) Widespread pain index (WPI) 7 and symptom severity (SS) scale score 5 or WPI 3–6 and SS scale score 9.

2) Symptoms have been present at a similar level **for at least 3 months.**

3) The patient does not have a disorder that would otherwise explain the pain.

Ascertainment

1) WPI: note the number areas in which the patient has had pain over the last week. In how many areas has the patient had

pain? Score will be between 0 and 19.

Shoulder girdle, left Hip (buttock, trochanter), left Jaw, left Upper back

Shoulder girdle, right Hip (buttock, trochanter), right Jaw, right Lower back

Upper arm, left Upper leg, left Chest Neck

Upper arm, right Upper leg, right Abdomen

Lower arm, left Lower leg, left

Lower arm, right Lower leg, right

2) SS scale score:

Fatigue

Waking unrefreshed

Cognitive symptoms

For the each of the 3 symptoms above, indicate the level of severity over the past week using the following scale:

0 no problem

1 slight or mild problems, generally mild or intermittent

2 moderate, considerable problems, often present and/or at a moderate level

3 severe: pervasive, continuous, life-disturbing problems

Considering somatic symptoms in general, indicate whether the patient has:*

0 no symptoms

1 few symptoms

2 a moderate number of symptoms

3 a great deal of symptoms

The SS scale score is the sum of the severity of the 3 symptoms (fatigue, waking unrefreshed, cognitive symptoms) plus the

extent (severity) of somatic symptoms in general. The final score is between 0 and 12.

* Somatic symptoms that might be considered: muscle pain, irritable bowel syndrome, fatigue/tiredness, thinking or remembering problem, muscle

weakness, headache, pain/cramps in the abdomen, numbness/tingling, dizziness, insomnia, depression, constipation, pain in the upper abdomen,

nausea, nervousness, chest pain, blurred vision, fever, diarrhea, dry mouth, itching, wheezing, Raynaud’s phenomenon, hives/welts, ringing in ears,

vomiting, heartburn, oral ulcers, loss of/change in taste, seizures, dry eyes, shortness of breath, loss of appetite, rash, sun sensitivity, hearing

difficulties, easy bruising, hair loss, frequent urination, painful urination, and bladder spasms.

1. **Chronic fatigue syndrome: the Revised Canadian Criteria**

In this study, we will apply the ICD-10 code G93.3 for ME/CFS.

A patient with ME/CFS will meet the criteria for fatigue, post-exertional malaise and/or fatigue, sleep dysfunction and pain; have two or more neurological/cognitive manifestations and one or more symptoms from two of the categories of (a) autonomic, (b) neuroendocrine and (c) immune manifestations; and adhere to item 7.

1. Fatigue

Required The patient must have a significant degree of new onset, unexplained, persistent, or recurrent physical and mental fatigue that substantially reduces activity level.

1. Post-Exertional Malaise and/or Post-Exertional Fatigue

Required There is an inappropriate loss of physical and mental stamina, rapid muscular and cognitive fatigability, post-exertional malaise and/or post-exertional fatigue and a tendency for other associated symptoms within the patient’s cluster of symptoms to worsen.

There is a pathologically slow recovery period – usually 24 hours or longer.

1. Sleep Dysfunction (*)

Required There is unrefreshing sleep or sleep quantity or rhythm disturbances such as reversed or chaotic diurnal sleep rhythms. Note that patients without sleep dysfunction can still meet the diagnostic criteria if their illness began with an infection — see (*) below.

1. Pain (*)

Required There is a significant degree of myalgia. Pain can be experienced in the muscles, and/or joints, and is often widespread and migratory in nature. Often there are significant headaches of new type, pattern or severity. Note that patients without pain can still meet the diagnostic criteria if their illness began with an infection — see (*) below.

1. Neurological / Cognitive Manifestations

Two or more Two or more of the following difficulties should be present:

Confusion

Impairment of concentration and short-term memory consolidation

Disorientation

Difficulty with information processing, categorizing and word retrieval (Word-finding problems)

Perceptual and sensory disturbances (for example spatial instability and disorientation and inability to focus vision)

Ataxia, muscle weakness and fasciculations are common. There may be overload1 phenomena: cognitive overload, sensory overload (for example photophobia and hypersensitivity to noise) and/or emotional overload, which may lead to crash2 periods and/or anxiety.

1. At Least One Symptom from Two of the Following Three Categories

(autonomic, neuroendocrine, immune) At Least One Symptom from Two of the Following Three Categories:

(a) Autonomic Manifestations

Orthostatic intolerance (either neurally mediated hypotension, postural orthostatic tachycardia syndrome or delayed orthostatic hypotension)

Light-headedness

Extreme pallor

Nausea and irritable bowel syndrome

Urinary frequency and bladder dysfunction

Heart palpitations with or without cardiac arrhythmias

Exertional dyspnea

(b) Neuroendocrine Manifestations

Loss of thermostatic stability (subnormal body temperature and marked diurnal fluctuation)

Sweating episodes, recurrent feelings of feverishness

cold extremities (cold hand and feet)

Intolerance of extremes of heat and cold

Marked weight change (anorexia or abnormal appetite)

Loss of adaptability and worsening of symptoms with stress

(c) Immune Manifestations

Tender lymph nodes

Recurrent sore throat

Recurrent flu-like symptoms

General malaise

New food sensitivities, medication sensitivities and/or chemical sensitivities

1. The Illness Persists for at Least Six Months

Required It usually has a distinct onset, (**) although it may be gradual. Preliminary diagnosis may be possible earlier. Three months is appropriate for children.

1. **Post Covid-19 condition**

The WHO definition of post COVID-19 condition is as follows:

‘Post Covid-19 condition is defined as the continuation or development of new symptoms 3 months after the initial SARS-CoV-2 infection, with these symptoms lasting for at least 2 months with no other explanation.’

In this study we require:

1. Verified SARS-CoV-19 infection either by PCR testing, antibodies or hospitalization
2. Debilitating symptoms lasting for more than three months
