# Supplementary material for: Effectiveness of mindfulness-based online therapy or internet-delivered cognitive behavioral therapy compared with treatment as usual among patients with persistent somatic symptoms: Protocol for a randomized controlled trial
Source: PLoS One. 2025 Feb 12;20(2):e0316169. doi: 10.1371/journal.pone.0316169 (PMC11819597; doi:10.1371/journal.pone.0316169)
Supplement: S2 Appendix — (PDF) [file pone.0316169.s003.pdf]

Power analysis for AIR and internet therapy versus treatment as usual randomized controlled trial

Analysis report

Mikko Venäläinen

7.6.2021

Abstract

This report describes power analysis performed for a study aiming at testing whether an Amygdala and insula retraining (AIR) program and internet therapy developed at the Helsinki University Hospital (HUS) are more effective in the treatment of functional disorders, fibromyalgia, long Covid and chronic fatigue syndrome when compared to treatment as usual (TAU). The study is planned to be carried out as a multi-center randomized controlled trial with study units recruited from the Network for Functional Disorders hosted by the HUS Clinic for Functional Disorders. It is hypothesized the AIR is not worse than the internet therapy. Alternatively, it is hypothesized that both the AIR program and the internet therapy are more effective than TAU. According to simulations carried out based on a previous report, **it was estimated that a sample size of at least 90 individuals per experimental group is required for reaching a statistical power of >80% for outcome measures demonstrating improvement in AIR patients as compared to treatment equivalent to relaxation therapy (RT). This sample size was sufficient for all 9 out of 9 previously studied outcome measures reflecting functional impact, clinical severity, pain catastrophizing, severity of anxiety and depressive symptoms, perceived health status, psychological inflexibility, and mindfulness at post-treatment and three-month follow-up**.

Background

Power analysis is an important aspect of experimental design used to determine the sample size required to detect an effect of a given size with a given degree of confidence. In principle, statistical power is the probability that the test correctly rejects the null hypothesis in statistical hypothesis testing. Therefore, the higher the statistical power, the higher the probability of detecting a statistically significant effect in the collected data. Typically, experiments are designed to have statistical power of at least 80%. For realistic estimation of statistical power with different sample sizes, prior information on the effect of interest under the planned statistical hypothesis testing from the literature or relevant pilot studies is required.

There is limited amount of information available for the comparison of AIR with other treatment strategies. However, Sanabria-Mazo et al. (2020) reported that mindfulness plus AIR demonstrated significantly greater reductions in functional impairment, anxiety, and depression, as well as higher improvements in mindfulness, and self-compassion at post-treatment and three-month follow-up, with moderate to large effect sizes as compared to RT in patients with fibromyalgia. The analyses were performed using hierarchical linear mixed-effects models, nesting participants at level 1 as random effects to control for the correlation between repeated measurements, and considering each outcome variable, on an intention-to-treat (ITT) basis.

The aim of this study was to perform power analyses for the statistical models and outcome measures used in Sanabria-Mazo et al. (2020) in order to facilitate planning of the sample sizes for the upcoming multi-center

randomized controlled trial organized by HUS. Despite the differences between the two studies, the present analyses can be used as a guideline for the expected number of samples required for detecting statistically significant differences in clinically relevant outcome measures between AIR and HUS internet therapy versus TAU.

Methods

All statistical analyses were performed using the R statistical computing environment version 4.0.3 (R Core Team, 2016. R: A language and environment for statistical computing. R Foundation for Statistical Computing, Vienna, Austria. URL https://www.R-project.org/). The power and sample size calculations were performed using the R package *simr* (version 1.0.5) that calculates power for generalized linear mixed models from the *lme4* package via Monte Carlo simulations. Similar to Sanabria-Mazo et al. (2020), the models were constructed to include nesting of participants as random effects in the form

$$Value \sim Group * Time + (1|Subject)$$

In order to simulate power estimates for AIR versus treatment equivalent to RT with varying sample sizes, prior information regarding model parameters is required. In Sanabria-Mazo et al. (2020), the regression coefficients have been given only for the $Group * Time$ interaction between the groups at post-intervention and follow-up assessments. Therefore, the missing regression coefficients were approximated from reported group means at different time points. The applied regression coefficients for different outcome measures are shown in table below.

Model parameter	FIQ	CGI-S	PCS	HADS-A	HADS-D	EQ-VAS	AAQ-II	FFMQ	SCS
Intercept	61.12	4.27	25.93	11.53	8.33	53.07	38.00	120.07	17.23
GroupAIR	6.91	0.20	4.20	0.89	0.99	-5.18	2.95	-2.91	-0.92
Time1	0.10	0.06	-2.46	-1.00	-0.80	3.80	1.07	1.80	-0.04
Time2	6.70	-0.20	-2.40	-1.73	-0.53	8.60	-1.00	2.60	-0.09
GroupAIR:Time1	-26.38	-0.72	-3.48	-3.15	-4.07	12.26	-7.64	13.44	3.80
GroupAIR:Time2	-23.99	-0.98	-10.00	-3.75	-3.66	14.08	-14.06	12.46	6.78

In addition, variance for random effects as well as residual standard deviation (SD) needed to be provided for the models. These were estimated via modeling with artificial data generated by drawing samples for AIR and RT groups with original sample sizes from normal distributions according to means and SDs reported in Sanabria-Mazo et al. (2020). Then, the linear mixed model was fitted to the artificial data to obtain an estimate for residual SD and random intercept. The data generation and model fitting was repeated 100 times to estimate full range of potential values. The simulation results are shown in tables below.

Residual SD	FIQ	CGI-S	PCS	HADS-A	HADS-D	EQ-VAS	AAQ-II	FFMQ	SCS
Minimum	13.40	0.8171	8.915	3.254	3.434	13.52	8.973	14.23	3.246
1st Quartile	16.29	0.9502	10.369	3.715	4.237	15.66	10.327	16.78	3.734
Median	17.07	0.9952	10.917	4.020	4.484	16.52	10.990	17.65	3.937
Mean	17.05	1.0050	10.867	4.002	4.496	16.54	11.059	17.74	3.954
3rd Quartile	18.01	1.0677	11.508	4.230	4.729	17.45	11.721	18.88	4.183
Maximum	21.15	1.2585	13.085	5.048	5.527	20.49	13.624	21.50	4.894

Random intercept	FIQ	CGI-S	PCS	HADS-A	HADS-D	EQ-VAS	AAQ-II	FFMQ	SCS
Minimum	0.000	0.0000	0.000	0.000	0.000	0.000	0.000	0.000	0.000
1st Quartile	0.000	0.0000	0.000	0.000	0.000	0.000	0.000	0.000	0.000

Random intercept	FIQ	CGI-S	PCS	HADS-A	HADS-D	EQ-VAS	AAQ-II	FFMQ	SCS
Median	0.000	0.0000	0.000	0.000	0.000	0.000	0.000	0.000	0.000
Mean	2.245	0.1394	1.458	0.5924	0.6342	2.3088	1.5305	2.4073	0.5357
3rd Quartile	4.550	0.2764	2.970	1.2532	1.2737	4.5788	3.0376	4.7060	1.0820
Maximum	8.547	0.5700	5.997	2.3214	2.3625	9.1463	6.1170	9.9938	2.1769

To avoid too optimistic power estimates, the maximum values estimated for residual SD and random intercept were used in power calculations for each outcome measure. The calculations were performed for sample sizes of 10, 20, 30, 40, 50, 60, 70, 80, 90 and 100 individuals per experimental group. For each sample size, a total of 100 simulations were performed.

Results

The results of power calculations for all outcome measures are shown in the figure below. The vertical blue lines visible in the figure indicate the minimum number of individuals per experimental group required for achieving a statistical power of >80% for comparisons between AIR and treatment equivalent to RT at post-treatment and three-month follow-up. It was estimated that a minimum of 90 individuals per group should be sufficient to achieve >80% power for all of the previously studied outcome measures. Excluding outcome measure PCS, a sample size of 70 individuals per group would be sufficient for all the other outcome measures.

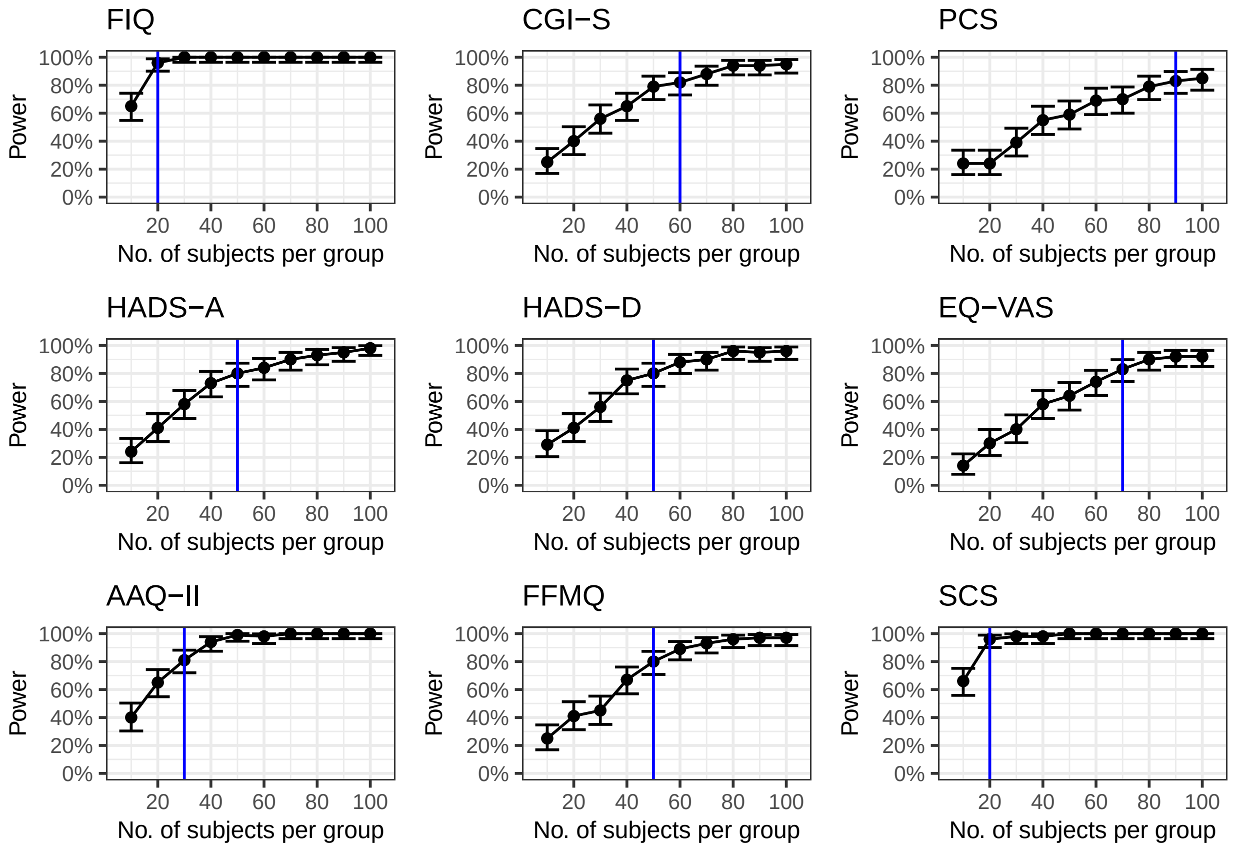

Discussion

Here, power calculations for the improvements in several outcome measures in AIR patients as compared to RT patients as observed in Sanabria-Mazo et al. (2020) were performed. The results suggested that a sample size of 90 individuals per experimental group would be sufficient for achieving a statistical power of >80% at for all previously studied outcome measures post-treatment and three-month follow-up.

Despite the clear result, it should be noted that the results are not necessarily directly applicable to the planned multi-center randomized controlled trial. In Sanabria-Mazo et al. (2020), all patients were women with fibromyalgia whereas the planned trial consists of patients with bodily stress syndrome, fibromyalgia, long Covid and chronic fatigue syndrome. In addition, there might be potential differences in given treatments, *e.g.* RT is not equivalent to TAU, and thus the effects might be different than assumed in the present analyses.

Most notably, however, the outcome measures between the two studies are also different and it is not guaranteed that effects of similar size will be seen in the outcome measures planned for the upcoming trial. Regardless, the given estimate for the minimum sample size ensured high power for all previously studied outcome measures even when greatest amount of error for the residual SD and random intercept was assumed. Therefore, under the assumption that the different outcome measures can all detect differences between the patient groups and that the effects are true, the demonstrated minimum sample size is likely to be sufficient for statistically significant findings. Finally, in the upcoming trial, data at even more follow-up time points is planned to be collected and thus even a smaller sample size than estimated here might actually be enough.

In conclusion, the present analysis demonstrate that a sample size of 90 individuals per experimental group might be sufficient in the planned multi-center randomized controlled trial. This result, even though calculated with maximum errors, should however be treated with caution as there are also several inherent differences between the two studies.

References

Sanabria-Mazo, Juan P., Jesus Montero-Marin, Albert Feliu-Soler, Virginia Gasión, Mayte Navarro-Gil, Héctor Morillo-Sarto, Ariadna Colomer-Carbonell, et al. 2020. "Mindfulness-Based Program Plus Amygdala and Insula Retraining (MAIR) for the Treatment of Women with Fibromyalgia: A Pilot Randomized Controlled Trial." *Journal of Clinical Medicine* 9 (10). https://doi.org/10.3390/jcm9103246.
