# Supplementary material for: Effectiveness of mindfulness-based online therapy or internet-delivered cognitive behavioral therapy compared with treatment as usual among patients with persistent somatic symptoms: Protocol for a randomized controlled trial
Source: PLoS One. 2025 Feb 12;20(2):e0316169. doi: 10.1371/journal.pone.0316169 (PMC11819597; doi:10.1371/journal.pone.0316169)
Supplement: S1 File — (PDF) [file pone.0316169.s004.pdf]

281121

Version 2.1

Helena Liira, Mari Kanerva, Teemu Mäntylä, Karoliina Hirvonen, Olli Kontulainen, Eemil Partinen, Markku Sainio, Iiris Kasanen, Anna-Mari Kiviniemi, Antti Malmivaara, Tiina Paunio, Jan-Henry Stenberg, Mikko Varonen, Aki Vuokko, Jari Arokoski

Amygdala and insula retraining (AIR) program and HUS internet therapy compared to treatment as usual in bodily stress syndrome, fibromyalgia, long Covid, and chronic fatigue syndrome (ME/CFS): A multi-center randomized controlled trial.

Abstract

Functional disorders are a challenging group of conditions treated both in primary and secondary care. Recently, mounting evidence shows that central nervous system sensitization is one of the involved mechanisms, giving new treatment opportunities. There are self-management programs and internet therapies that aim at overcoming the central sensitization by creating new neural networks in the brain. This study aims to test whether an Amygdala and insula retraining (AIR) program compared to an internet therapy developed at HUS (Helsinki University Hospital) and treatment as usual (TAU) is effective in the treatment of functional disorders, fibromyalgia, long Covid, and chronic fatigue syndrome (ME/CFS).

We will perform a multi-center randomized controlled trial in volunteering health units and with volunteering patients aiming at 360 patients altogether. The physicians in charge of the patients will be recruited from the Network for Functional Disorders launched by the novel clinic at HUS. The participating centers will receive an introduction in functional disorders, the underlying mechanisms, and treatment opportunities.

For functional disorders, this study applies the ICD-11 primary care code of Bodily stress syndromes (BSS), which covers fibromyalgia, irritable bowel syndrome, multiple chemical sensitivities, and other, both single system and multiorgan functional disorders. In addition, for patients with fibromyalgia, chronic fatigue symptoms, and long Covid, we will apply specific diagnostic criteria. Both primary and secondary care treatment units that manage these disorders can recruit patients.

The studied interventions are a mindfulness-based Amygdala and insula retraining (AIR) program and an internet therapy developed at HUS, and patients receive usual care in addition to these interventions. The third study arm consists of treatment as usual for six months after which the patients receive the AIR program or the internet therapy if they so wish. When the participating physicians consider that a patient could benefit from a self-management program, they introduce the trial to the patient. The consenting patient fills in a baseline survey and is followed up at 3, 6, and 12 months with the help of the HUSeCRF (online surveys at the HUS electronic Case Report Form), or research nurse's interviews.

This study will give new insight into the opportunities of self-management programs in the treatment of bodily stress syndromes, fibromyalgia, long Covid, and ME/CFS. We will find out whether the training programs increase patients' functional ability and quality of life as measured on a scale from 0 to 10. These scales and functional ability as measured by WHODAS 2.0 serve as main outcome measures for this study. This trial is the first large-scale randomized trial that assesses

these methods developed to overcome the central sensitization of brain. The results will have an impact on the management of these common and often debilitating conditions.

I **Background**

Functional disorders are conditions where a patient is troubled by physical symptoms that cannot be attributed to any known physical or psychiatric disorder (Budtz-Lilly et al., 2015). These conditions have long been known as medically unexplained symptoms. However, mounting evidence shows that the central nervous system has a significant role in these problems, and recently they have been referred to as central sensitization syndromes. There is a novel definition for these conditions in the primary care version of the WHO ICD-11 coding: bodily stress syndromes (BSS) that we will apply in this research plan (Goldberg et al., 2016, Lam et al., 2013). In addition, we will include patients that fulfill the specific diagnostic criteria of fibromyalgia, chronic fatigue syndrome, and long Covid (Wolfe et al., Jason et al., 2010, Dani et al., 2021), all of which have elements of dysfunctional autonomous nervous system.

A novel challenge is long Covid, which is a syndrome characterized by debilitating symptoms (including breathlessness, chest pain, palpitations, and orthostatic intolerance) which can last for months after the acute illness (Dani et al., 2021, Iqbal et al., 2021). It has been hypothesized that the cytokine storm, organ damage by the virus, immunologic mechanisms and autoimmunity, as well as consequences to the autonomous nervous system are among the biological factors that may contribute to persistent symptoms (Proal and Van Elzakker, 2021). According to the latest estimates around 14% of patients with confirmed infection report symptoms after 12 weeks of first symptoms (Office for National Statistics, UK, 2021).

Most likely, the origin of functional syndromes (Kato et al. 2010) and long Covid is multifactorial and includes an interaction of biological, psychological, and cultural factors. There is often predisposed vulnerability related to genetic or developed traits and attitudes, triggering, and maintaining factors in these conditions. Usually, the onset is a consequence of stress, trauma, or a stressful biological factor such as a viral infection. Because of the multi-factorial origin, it has been challenging to create effective treatment modalities for these disorders.

Treating medically unexplained syndromes is considered challenging by physicians. Bodily stress syndromes are common, and it has been estimated in Denmark that 16% of the adult population suffer from them (Petersen et al., 2020). Clinics for functional disorders, psychosomatics, or central sensitization syndromes are opened in many countries to develop the care of these disorders. At Helsinki University Hospital (HUS), such a clinic was opened in 2019.

Many treatment options are based on cognitive behavioral therapy (CBT). A Cochrane review assessed psychological therapies, especially CBT, in medically unexplained symptoms (van Dessel et al., 2014). They were superior to usual care or waiting list in terms of reduction of symptom severity, but effect sizes were small. Similarly, another Cochrane review found that CBT is effective in reducing the symptoms of chronic fatigue syndrome (CFS) at post-treatment compared with usual care, and may be more effective in reducing fatigue symptoms compared with other psychological therapies (Price et al., 2008).

However, CBT is not widely available and patients suffering from functional disorders are many. More readily available non-drug based therapies are needed. There is some evidence that

neuroplasticity techniques developed by those suffering from these conditions may be effective in the treatment of CFS (Gupta 2002, Gupta 2010) and fibromyalgia (Sanabria-Mazo et al., 2020). In addition, there is some evidence that CBT administered as internet therapy is effective in CFS (Worm-Smeitink et al., 2020).

This study will compare an Amygdala and insula retraining (AIR) program to an internet therapy developed at HUS. The study will be a multi-center randomized controlled trial. Study units will be recruited from the Network for Functional Disorders that is hosted by the HUS Clinic for Functional Disorders and potentially later from international collaborators. The trial will clarify whether internet-based non-drug based therapies are helpful in overcoming the central sensitization and the functional disabilities caused by these disorders.

II The aim of the study

We aim to explore what is the effect of the AIR program and an Internet therapy on patients' assessment of functional ability and quality of life on a scale from 0 to 10 and functional ability measured by WHODAS 2.0 as compared to treatment as usual at six months.

The first hypothesis is that the AIR is not worse than the internet therapy in the management of bodily stress syndromes, fibromyalgia, and chronic fatigue syndrome. Another hypothesis is that both the AIR program and the internet therapy are more effective than treatment as usual.

III Research plan and methods

Material and methods

Inclusion criteria:

Age 18 to 65 years

- The criteria of bodily stress syndrome, fibromyalgia, chronic fatigue syndrome, or long Covid fulfilled (See Attachment 1) AND
- Diagnostic examinations have ruled out the potential somatic reasons for the symptoms AND
- Disabling symptoms have lasted at least 3 months AND
- Patient is willing to receive a psychoeducation or brain retraining intervention.

Exclusion criteria:

- Patients for whom participation could be overly demanding because of physical constraints (for example, patients who cannot write because of dystonia or bedridden patients)
- Patients with presence of severe psychiatric and severe somatic disorders, e.g. ***moderate or severe depression or*** newly onset cancer for which the study could be overly strenuous.

Interventions

- The **Amygdala and insula retraining program** consists of novel brain retraining approaches focused on hypothetically interrupting and retraining the conditioned defensive hyper-stimulation of the sympathetic nervous system and aspects of the immune system by the amygdala and insula, to bring the brain and body back to homeostasis. It includes supportive

techniques such as breathing, meditation, and neurolinguistic programming. The patients are invited to an online program of video session and eight weekly 2 h webinar sessions followed by three monthly sessions. The patients are assigned to do daily homework that takes approximately 15 to 20 min to complete

- The **HUS internet therapy for bodily stress syndromes (iHUSbss)** includes psychoeducation about autonomic nervous system and of the effects of patient's own thinking and action on the nervous system. The exercises aim at relaxing the body, at novel ways to observe the symptoms, and at developing acceptance and self-compassion. The program includes exercises that are done regularly in everyday life.
- The control group consists of treatment as usual for six months. After six months the patients are invited to take part in either the AIR program or the Internet therapy if they so wish.

Treatment as usual

In addition to study intervention, patients receive treatment as usual (TAU). The patients may have appointments with their physician, physiotherapist, or another health professional, and they may attend group interventions. If possible, medication is kept the same during the intervention and three months afterwards. If there is need to change the medication during the trial, the changes will be recorded. Appointments with health professionals and attendance in group interventions are monitored.

Study flow

In the beginning, the patient receives written information and an oral explanation of the study from the physician and is asked to give written informed consent. Patients have the right to withdraw from the study at any point if they so wish with no consequences to their treatment.

The patients ***are asked to give their consent to the study via Suomi.fi-service.*** They then fill in ***online*** the baseline information form. ~~and sign the informed consent, which are sent to the study base~~. The research assistant randomizes the patient in the HUSeCRF-system (Helsinki University Hospital electronic Case Report Form)~~. The study centers are provided with blocks of randomized interventions (block size varies) in sealed envelopes. The randomized interventions are used consecutively. If the patient consents to the study, the physician opens an envelope, which contains the information for~~ and sends the patient information for the follow-up, username and password to enter follow up data in the HUSeCRF system.

Follow-up

The follow up is primarily performed with the HUSeCRF system. The patient receives reminders either by phone or via email when it is time to fill in the 3, 6 and 12 months follow up surveys. If the patients lose their username or passcode, the study group sends new ones as needed. If the patient has not filled in the questionnaires after two reminders, the study nurse telephones the patient and interviews the outcomes via telephone.

Outcomes

The main outcomes in the study are functional ability and quality of life as measured on a scale from 0 to 10 and functional ability measured by the WHODAS instrument at six months. All outcomes of the study are presented in Table 1.

Table 1. Outcomes of the RCT and the timepoints of measurement

	Baseline, 0 mo	3 mo	**6 mo**	12 mo
**Main outcome measures**				
Functional ability 0 - 10	x	x	**x**	x
Quality of life 0 - 10	x	x	**x**	x
Functional ability: WHODAS2.0	x	x	**x**	x
**Other outcome measures**				
Baseline information: other chronic diseases, medications, social status, occupation	***x***			
Follow-up questionnaire (PREMs, treatments, feedback)		x	x	x
Quality of life: EuroHIS-QOL-8 (Power 2003)	x	x	x	x
Health related Quality of life: 15D (Sintonen 2001)	x	x	x	x
Symptoms, SSD-12 (Toussaint et al., 2016) and PHQ-15 (Kroenke et al., 2002)	x	x	x	x
Depression, PHQ-9 (Kroenke et al., 2001)	x	x	x	x
Anxiety, GAD-7 (Spitzer et al., 2006)	x	x	x	x
ISI, Insomnia Severity Index (Morin et al., 2011)	x	x	x	x
Work ability (subjective, three steps) (Health 2000 Study Group)	x	x	x	x
Resilience Resilience Scale-14 (RS-14) (Wagnild 2009)	x	x	x	x
Researcher collects information from patient health record: diagnoses, medications and other possible treatments during 12 months				***x***

To collect clinical information, a member of the research group will access the patient health record system at 12 months to collect information on diagnoses, medications, and other possible treatments during the follow up.

Data handling, power calculations and statistical methods

A research assistant will store the baseline data in pseudonymized format into the HUSeCRF database. Access to the database is given to those members of the research group that analyze the data. Access to HUSeCRF is with personal codes and includes a telephone verification.

The codes that link patient information and study numbers are saved in the Key register, the access of which only the PI and the study coordinators have.

Power calculations (see the attachment): It is hypothesized the AIR is not worse than the internet therapy. Alternatively, it is hypothesized that both the AIR program and the internet therapy are more effective than TAU. According to simulations carried out based on a previous report, it was estimated that a sample size of at least 90 individuals per experimental group is required for reaching statistical power of >80% for outcome measures demonstrating improvement in AIR patients as compared to treatment equivalent to relaxation therapy (RT). This sample size was sufficient for all 9 out of 9 previously studied outcome measures reflecting functional impact, clinical severity, pain catastrophizing, severity of anxiety and depressive symptoms, perceived health status, psychological inflexibility, and mindfulness at post-treatment and three-month follow up. To overcome loss to follow up, we aim at 120 patients per each treatment arm.

IV Timetable

The research plan will be presented to the Ethics committee at Helsinki University Hospital in summer 2021. The study will be started as soon as participating health professionals have got the introduction and the plan has been accepted in HUS ethics board and in the ethics boards of the study units.

7/2021 9/2021 12/2021 3/2021 6/2022 9/2022 12/2022

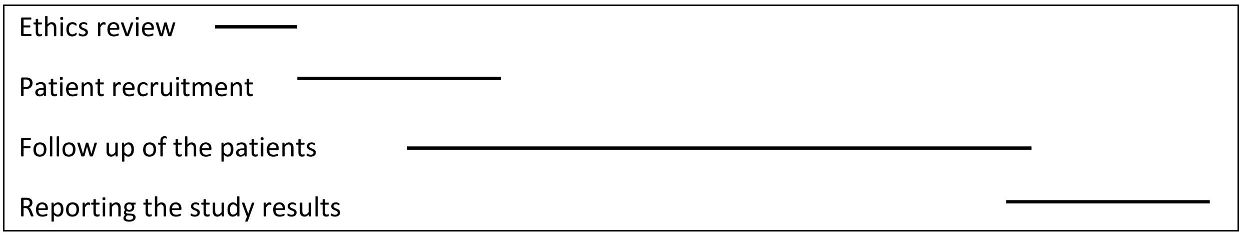

V Research group and tasks

The research group consists of researchers and clinicians that work at the Clinics for Functional Disorders and Long Covid at Helsinki University Hospital. The principal investigator (PI) (Liira) has previous experience of conducting randomized controlled trials. The vice PI Prof Arokoski has vast experience in clinical trials. Drs Kasanen, Paunio, Sainio and Vuokko have previously conducted research on functional disorders in Finland. Prof Malmivaara has helped the group as methods expert. In addition, health care professionals in the study centers are included in the research group, and they have taken part in the planning of the study.

VI Research funding

Dr Liira received a State Research Fund grant of 100,000 euros, and there is a permission from the research lead that this grant can be used in this study, as soon as the ethics process and research permission process are finalized. Further funding will be searched for this trial.

VII Ethical aspects

This study is conducted in voluntary health care units with voluntary patients. For patients, it means an opportunity to receive an additional self-management program and possibly get help for the symptoms they suffer from. Taking part in a study means filling in forms, which can be time consuming. In addition to the self-management program, the trial does not change the treatment the patient receives. Patients have the right to withdraw from the study at any point if they so wish and in this case their information is no longer included in the study.

VIII The importance of the study for the Helsinki University Hospital

Up to 8% of secondary care patients suffer from functional disorders, and the care of this patient group generally is not well organized. This study will explore non-drug based self-management interventions that are available to patients both in primary and secondary care. This trial is the first large-scale randomized trial that assesses the methods developed to overcome the central sensitization of brain. The results will develop the management of these common and often debilitating conditions. Applying these methods may save costs as patients use less health care services when they have less symptoms.

References

Dani M, Dirksen A, Taraborrelli P, Torocastro M, Panagopoulos D, Sutton R, Lim PB. Autonomic dysfunction in 'long COVID': rationale, physiology and management strategies.Clin Med (Lond). 2021 Jan;21(1):e63-e67.

Goldberg D, Reed R, Robles R, et al. Multiple somatic symptoms in primary care: A field study for ICD-11 PHC, WHO's revised classification of mental disorders in primary care settings. J Psychosom Res 2016; 91: 48-54.

Gupta A. Unconscious amygdalar fear conditioning in a subset of chronic fatigue syndrome patients. Medical Hypotheses 2002; 50 (6): 727-35.

Gupta A. Can amygdala retraining techniques improve the wellbeing of patients with chronic fatigue syndrome.

Health 2000 in brief. Available at: https://thl.fi/en/web/thlfi-en/research-and-expertwork/projects-and-programmes/health-2000-2011/health-2000-in-brief (accessed 29 Nov 2020).

Houwen J, Lucassen PLBJ, Stappers HW, Assendelft PJJ, van Dulmen S, Olde Hartman TC. Medically unexplained symptoms: the person, the symptoms and the dialogue. Fam Pract. 2017;34(2):245-251.

Iqbal FM, Lam K, Sounderajah V, Clarke JM, Ashrafian H, Darzi A. Characteristics and predictors of acute and chronic post-COVID syndrome: A systematic review and meta-analysis. EClinicalMedicine 2021; 36: 100899. doi: doi.org/10.1016/j.eclinm.2021.100899.

Jason L, Evans M, Porter N, et al. The development of a revised Canadian myalgic encephalomyelitis chronic fatigue syndrome case definition. Am J Biochem Biotechnol 2010;6:120-35

Kato K, Sullivan P, Pedersen N. Latent class analysis of functional somatic symptoms in a population-based sample of twins. J Psychosom Res. 2010 May;68(5):447-53. doi: 10.1016/j.jpsychores.2010.01.010. Epub 2010 Mar 1.

Kroenke K, Spitzer RL, Williams JB. The PHQ-9: validity of a brief depression severity measure. J Gen Intern Med. 2001 Sep;16(9):606-13.

Kroenke K, Spitzer RL, Williams JB. The PHQ-15: validity of a new measure for evaluating the severity of somatic symptoms. Psychosom Med 2002;64(2):258-66.

Morin CM, Belleville G, Bélanger L, Ivers H. The Insomnia Severity Index: psychometric indicators to detect insomnia cases and evaluate treatment response. Sleep 2011;34:601-8.

Lam T, Goldberg D, Dowell A, et al. Proposed new diagnoses of anxious depression and bodily stress syndrome in ICD-11-PHC: an international focus group study. Fam Pract 2013; 30 (1): 76–87.

Office for National Statistics, UK. Prevalence of ongoing symptoms following coronavirus (COVID-19) infection in the UK: 1 April 2021, https://www.ons.gov.uk/peoplepopulationandcommunity/healthandsocialcare/conditionsanddiseases/bulletins/prevalenceofongoingsymptomsfollowingcoronaviruscovid19infectionintheuk/1april2021 (retrieved 24th June, 2021).

Petersen MW, Schröder A, Jørgensen T, Ørnbøl E, Meinertz Dantoft T, Eliasen M, Benros ME, Fink P. Irritable bowel, chronic widespread pain, chronic fatigue and related syndromes are prevalent and highly overlapping in the general population: DanFunD. A register-based case-control study. Sci Rep. 2020;10(1):3273.

Power M. Development of a common instrument for quality of life. In: Nosikov A, Gudex C. (eds). EUROHIS: Developing Common Instruments for Health Surveys. IOS Press, Amsterdam, the Netherlands. 2003. Pages 145–159.

Price JR, Mitchell E, Tidy E, Hunot V. Cognitive behaviour therapy for chronic fatigue syndrome in adults. Cochrane Database of Systematic Reviews 2008, Issue 3. Art. No.: CD001027. DOI: 10.1002/14651858.CD001027.pub2. Accessed 25 March 2021.

Proal A, Van Elzakker M. Long COVID or post-acute sequelae of COVID-19 (PASC): An overview of biological factors that may contribute to persistent symptoms. Front Microbiol. 2021 Jun 23;12:698169. doi: 10.3389/fmicb.2021.698169. eCollection 2021.

Rosendal M. MUS becomes Bodily Stress Syndrome in the ICD-11 for primary care, Results from the WHO Primary Care Consultation Group on mental health. Available at: https://www.vumc.nl/afdelingen-themas/49661/20678990/4.3_Rosendal_MUS_BSS_WHO.pdf (accessed 29 Nov 2020).

Sanabria-Mazo JP, Montero-Marin J, Feliu-Soler A, et al. Mindfulness-based program plus amygdala and insula retraining (MAIR) for the treatment of women with fibromyalgia: A pilot randomized controlled trial. J Clin Med. 2020 Oct 11;9(10):3246. doi: 10.3390/jcm9103246.

Sintonen H. The 15D instrument of health-related quality of life: properties and applications. Ann Med 2001;33:328-36

Spitzer RL, et al. A brief measure for assessing generalized anxiety disorder: the GAD-7. Arch Intern Med. 2006;166(10):1092-7.

Taquet M, Geddes J, Husain M et al. 6-month neurological and psychiatric outcomes in 236 379 survivors of COVID-19: a retrospective cohort study using electronic health records. Lancet Psychiatry 2021, Published Online April 6, 2021. https://doi.org/10.1016/S2215-0366(21)00084-5

Toussaint A, Murray AM, Voigt K, Herzog A, Gierk B, Kroenke K et al. Development and validation of the somatic symptom disorder-B criteria scale (SSD-12). Psychosom Med 2016; 78(1):5-12.

Trollund Rask M, Ørnbøl E, Rosendal M, Fink P. Long-term outcome of bodily distress syndrome in primary care: A follow-up study on health care costs, work disability, and self-rated health. Psychosom Med 2017; 79 (3): 345-57.

Wagnild G. A review of the resilience scale. *J Nurs Meas* 2009; 17: 105–113.

van Dessel N, den Boeft M, van der Wouden JC, Kleinstäuber M, Leone SS, Terluin B, Numans ME, van der Horst HE, van Marwijk H. Non-pharmacological interventions for somatoform disorders and medically unexplained physical symptoms (MUPS) in adults. Cochrane Database of Systematic Reviews 2014, Issue 11. Art. No.: CD011142. DOI: 10.1002/14651858.CD011142.pub2. Accessed 25 March 2021.

Wolfe F, Clauw DJ, Fitzcharles MA, Goldenberg DL, Katz RS, Mease P, Russell AS, Russell IJ, Winfield JB, Yunus MB. The American College of Rheumatology preliminary diagnostic criteria for fibromyalgia and measurement of symptom severity. Arthritis Care Res (Hoboken). 2010 May;62(5):600-10. doi: 10.1002/acr.20140. PMID: 20461783

Worm-Smeitink M, Janse A, van Dam A, Evers A, van der Vaart R, Wensing M, Knoop H.Internet-based cognitive behavioral therapy in stepped care for chronic fatigue syndrome: Randomized noninferiority trial. J Med Internet Res. 2019 Mar; 21(3): e11276. Published online 2019 Mar 14. doi: 10.2196/11276

Appendix:

Diagnostic criteria

1. **Bodily stress syndrome (BSS): ICD-11-PHC**

In this study, since ICD-11 is not yet in use in Finland, for bodily stress syndrome, we will apply the ICD-10 code R68.88. This code is used, if the specific codes for ME/CFS, fibromyalgia, or long Covid are not fulfilled.

Presenting symptoms/complaints

The patient presents with multiple somatic symptoms over time in association with high distress, and accompanied by disability. The symptoms may be influenced by culture and change over time.

Clinical description

The patient suffers from multiple persistent bodily symptoms, which are present at the same time. In order to diagnose BSS, the symptoms must at some stage present as autonomic arousal symptoms, musculoskeletal tension or general/neurological and cognitive symptoms and result in significant disruption in daily life. Symptoms are distressing and/or result in significant disruption in daily life, as well as persistent concerns about the medical seriousness of the symptoms.

Required symptoms:

The patient must have

- At least 3 persistent symptoms over time attributable to autonomic over-arousal (cardio-respiratory, gastrointestinal, musculoskeletal) or as general symptoms of tiredness and exhaustion
- The patient's concern over health expresses itself as excessive time and energy devoted to these symptoms
- The symptoms are distressing and result in significant disability

Symptom patterns may include:

- Examples of cardiopulmonary arousal: palpitations, precordial discomfort, breathlessness without exertion, hyperventilation, hot or cold sweats, trembling or shaking, dry mouth
- Examples of gastrointestinal arousal: abdominal pains, frequent loose bowel movements, feeling bloated, regurgitations, constipation, diarrhoea, nausea, vomiting, burning sensation in chest or epigastrium
- Examples of musculoskeletal tension: pains in arms or legs, muscular aches or pains, pains in the joints, feelings of paresis or localized weakness, back ache, pain moving from one place to another, unpleasant numbness or tingling sensations
- Examples of general unspecific symptoms: concentration difficulties, impairment of memory, excessive fatigue, headache, dizziness

Exclusion:

- Those with anxiety or depression at case level should not be diagnosed as BSS, but sub-threshold anxious depression may be present. If the symptoms are accounted for by a known physical disease this is not BSS.

Severity

Mild: The patient complains of symptoms or problems in only one bodily system, and while there is some disability most activities can be managed, with increased difficulty.

Moderate: There are multiple problems in one or two bodily systems, and there is marked distress or disability associated with the symptoms.

Severe: There are symptoms in multiple bodily systems and disability/distress is severe.

Childhood variations

Bodily distress in children may be mono-symptomatic, and the type of symptoms varies with age, with abdominal pain and headache common in smaller children, whereas the prevalence of fatigue and neurological symptoms seems to increase with age. Bodily distress in children may continue into adult life.

Differential diagnosis

Consider physical disease with multiple symptoms, e.g. multiple sclerosis, hyperparathyroidism, acute intermittent porphyria, myasthenia gravis, AIDS, systemic lupus erythematosus, Lyme disease, connective tissues disease.

Psychiatric disorder with physical symptom presentation, e.g. substance use disorders, psychotic disorders.

Health anxiety if health concerns predominate rather than the symptoms themselves.

Conversion disorder if the symptom picture is dominated of neurological symptoms and the onset of symptoms is related to a severe psychological trauma.

2. Fibromyalgia: The American College of Rheumatology diagnostic criteria for fibromyalgia

In this study, we will apply the ICD-10 code M79.7 for fibromyalgia.

Criteria
A patient satisfies diagnostic criteria for fibromyalgia if the following 3 conditions are met:
1) Widespread pain index (WPI) 7 and symptom severity (SS) scale score 5 or WPI 3–6 and SS scale score 9.
2) Symptoms have been present at a similar level **for at least 3 months.**
3) The patient does not have a disorder that would otherwise explain the pain.
Ascertainment
1) WPI: note the number areas in which the patient has had pain over the last week. In how many areas has the patient had
pain? Score will be between 0 and 19.
Shoulder girdle, left Hip (buttock, trochanter), left Jaw, left Upper back
Shoulder girdle, right Hip (buttock, trochanter), right Jaw, right Lower back
Upper arm, left Upper leg, left Chest Neck
Upper arm, right Upper leg, right Abdomen
Lower arm, left Lower leg, left
Lower arm, right Lower leg, right
2) SS scale score:
Fatigue
Waking unrefreshed
Cognitive symptoms
For the each of the 3 symptoms above, indicate the level of severity over the past week using the following scale:
0 no problem
1 slight or mild problems, generally mild or intermittent
2 moderate, considerable problems, often present and/or at a moderate level

3 severe: pervasive, continuous, life-disturbing problems
Considering somatic symptoms in general, indicate whether the patient has:*
0 no symptoms
1 few symptoms
2 a moderate number of symptoms
3 a great deal of symptoms
The SS scale score is the sum of the severity of the 3 symptoms (fatigue, waking unrefreshed, cognitive symptoms) plus the
extent (severity) of somatic symptoms in general. The final score is between 0 and 12.
* Somatic symptoms that might be considered: muscle pain, irritable bowel syndrome, fatigue/tiredness, thinking or remembering problem, muscle
weakness, headache, pain/cramps in the abdomen, numbness/tingling, dizziness, insomnia, depression, constipation, pain in the upper abdomen,
nausea, nervousness, chest pain, blurred vision, fever, diarrhea, dry mouth, itching, wheezing, Raynaud's phenomenon, hives/welts, ringing in ears,
vomiting, heartburn, oral ulcers, loss of/change in taste, seizures, dry eyes, shortness of breath, loss of appetite, rash, sun sensitivity, hearing
difficulties, easy bruising, hair loss, frequent urination, painful urination, and bladder spasms.

3. Chronic fatigue syndrome: the Revised Canadian Criteria

In this study, we will apply the ICD-10 code G93.3 for ME/CFS.

A patient with ME/CFS will meet the criteria for fatigue, post-exertional malaise and/or fatigue, sleep dysfunction and pain; have two or more neurological/cognitive manifestations and one or more symptoms from two of the categories of (a) autonomic, (b) neuroendocrine and (c) immune manifestations; and adhere to item 7.

1. Fatigue

Required The patient must have a significant degree of new onset, unexplained, persistent, or recurrent physical and mental fatigue that substantially reduces activity level.

2. Post-Exertional Malaise and/or Post-Exertional Fatigue

Required There is an inappropriate loss of physical and mental stamina, rapid muscular and cognitive fatigability, post-exertional malaise and/or post-exertional fatigue and a tendency for other associated symptoms within the patient's cluster of symptoms to worsen.

There is a pathologically slow recovery period – usually 24 hours or longer.

3. Sleep Dysfunction (*)

Required There is unrefreshing sleep or sleep quantity or rhythm disturbances such as reversed or chaotic diurnal sleep rhythms. Note that patients without sleep dysfunction can still meet the diagnostic criteria if their illness began with an infection — see (*) below.

4. Pain (*)

Required There is a significant degree of myalgia. Pain can be experienced in the muscles, and/or joints, and is often widespread and migratory in nature. Often there are significant headaches of new type, pattern or severity. Note that patients without pain can still meet the diagnostic criteria if their illness began with an infection — see (*) below.

5. Neurological / Cognitive Manifestations

Two or more Two or more of the following difficulties should be present:

Confusion

Impairment of concentration and short-term memory consolidation

Disorientation

Difficulty with information processing, categorizing and word retrieval (Word-finding problems)

Perceptual and sensory disturbances (for example spatial instability and disorientation and inability to focus vision)

Ataxia, muscle weakness and fasciculations are common. There may be overload1 phenomena: cognitive overload, sensory overload (for example photophobia and hypersensitivity to noise) and/or emotional overload, which may lead to crash2 periods and/or anxiety.

6. At Least One Symptom From Two of the Following Three Categories

(autonomic, neuroendocrine, immune) At Least One Symptom From Two of the Following Three Categories:

(a) Autonomic Manifestations

Orthostatic intolerance (either neurally mediated hypotension, postural orthostatic tachycardia syndrome or delayed orthostatic hypotension)

Light-headedness

Extreme pallor

Nausea and irritable bowel syndrome

Urinary frequency and bladder dysfunction

Heart palpitations with or without cardiac arrhythmias

Exertional dyspnea

(b) Neuroendocrine Manifestations

Loss of thermostatic stability (subnormal body temperature and marked diurnal fluctuation)

Sweating episodes, recurrent feelings of feverishness

cold extremities (cold hand and feet)

Intolerance of extremes of heat and cold

Marked weight change (anorexia or abnormal appetite)

Loss of adaptability and worsening of symptoms with stress

(c) Immune Manifestations

Tender lymph nodes

Recurrent sore throat

Recurrent flu-like symptoms

General malaise

New food sensitivities, medication sensitivities and/or chemical sensitivities

7. The Illness Persists for at Least Six Months

Required It usually has a distinct onset, (**) although it may be gradual. Preliminary diagnosis may be possible earlier. Three months is appropriate for children.

4. Long Covid

There is no international consensus yet for the diagnostic criteria for long Covid. In this study we will apply the ICD-10 code U08.9 and the following diagnostic criteria:

1. Verified SARS-CoV-19 infection either by PCR testing, antibodies or hospitalization
2. Debilitating symptoms for more than three months
