# Supplementary material for: Effectiveness of mindfulness-based online therapy or internet-delivered cognitive behavioral therapy compared with treatment as usual among patients with persistent somatic symptoms: Protocol for a randomized controlled trial
Source: PLoS One. 2025 Feb 12;20(2):e0316169. doi: 10.1371/journal.pone.0316169 (PMC11819597; doi:10.1371/journal.pone.0316169)
Supplement: S2 File — (PDF) [file pone.0316169.s005.pdf]

Study title:	**Amygdala and insula retraining (AIR) program and HUS internet therapy compared to treatment as usual in bodily stress syndrome, fibromyalgia, long Covid, and chronic fatigue syndrome (ME/CFS): A multi-center randomized controlled trial. AIR and HUS net therapy trial**
Study Protocol:	Approval December 8, 2021
Reference number:	HUS/2239/2021

Helsinki, November 28, 2023

To whom it may concern,

The above referred protocol and related study materials were reviewed and approved by the HUS Ethics Committee, Finland. The original, official recommendation was given on December 8th, 2021.

Project timeline: 2022/01/01–2024/12/31

Doc. Helena Liira (Helsinki University Hospital) is listed as a principal investigator in the study protocol.

Faithfully yours,

Anna Pallari
Secretary of the Committee
Tel: +358 50 428 7386
