# Supplementary material for: Effectiveness of mindfulness-based online therapy or internet-delivered cognitive behavioral therapy compared with treatment as usual among patients with persistent somatic symptoms: Protocol for a randomized controlled trial
Source: PLoS One. 2025 Feb 12;20(2):e0316169. doi: 10.1371/journal.pone.0316169 (PMC11819597; doi:10.1371/journal.pone.0316169)
Supplement: Attachment — Submitted filename: Response to reviewers.docx [file pone.0316169.s006.docx]

| COMMENT | AMENDMENT |
| --- | --- |
| Please ensure that your manuscript meets PLOS ONE's style requirements, including those for file naming. | Thank you for this kind note.  We have checked the style guide and made corrections to the following naming of files/captions.  Fig 1. SPIRIT schedule of enrollment, interventions and assessments  Fig 2. Study design  S1. File. SPIRIT 2013 Checklist  S2 Appendix. Diagnostic criteria  S2 Appendix. Power Analysis  S4 Protocol Approved by Ethics Committee (English)  S5 Ethical Statement (HUS22392021) |
| We note that you have provided funding information that is currently declared in your Funding Statement. However, funding information should not appear in the Acknowledgments section or other areas of your manuscript. We will only publish funding information present in the Funding Statement section of the online submission form.  Please remove any funding-related text from the manuscript and let us know how you would like to update your Funding Statement. Currently, your Funding Statement reads as follows:  “This work is supported by European Union's Horizon Europe research and innovation programme (Long Covid HEU Grant no. 101057553 2022-2026) and State REsearch Funds. The funding sources have no other role in the study.”  Please include your amended statements within your cover letter; we will change the online submission form on your behalf. | Thank you for noticing this. We have removed funding information from the manuscript and mention it now in the cover letter:  ‘This work is supported by European Union's Horizon Europe Research and Innovation Programme (Long Covid, EU Grant no. 101057553 2022-2026) and State Research Funds. The funding sources have no other role in the study.’  Please ensure that it will be mentioned in the Funding Statement section. |
| When completing the data availability statement of the submission form, you indicated that you will make your data available on acceptance. We strongly recommend all authors decide on a data sharing plan before acceptance, as the process can be lengthy and hold up publication timelines. Please note that, though access restrictions are acceptable now, your entire data will need to be made freely accessible if your manuscript is accepted for publication. This policy applies to all data except where public deposition would breach compliance with the protocol approved by your research ethics board. If you are unable to adhere to our open data policy, please kindly revise your statement to explain your reasoning and we will seek the editor's input on an exemption. Please be assured that, once you have provided your new statement, the assessment of your exemption will not hold up the peer review process. | Thank you for pointing out these details.  Below is our revised data availability statement:  Data sharing is not applicable to this article as no datasets were generated or analyzed during the current study. |
| Please ensure that you refer to Figure 1 in your text as, if accepted, production will need this reference to link the reader to the figure. | Thank you for noticing this. Please see addition on line 185. |
| Please include captions for your Supporting Information files at the end of your manuscript, and update any in-text citations to match accordingly | Please see amendments for the first comment above. We have now included captions for our supporting information at the end of the manuscript and updated our in-text citations. |
| **REVIEWER COMMENTS** |  |
| **REV #1:** |  |
| 1- Line 277: Consider replacing “numbers” with “counts”. | Thank you for this suggestion. We have changed the wording. Please see line 299 |
| 2- Line 281: Indicate the type of underlying covariance structure that will be used in the linear mixed model or the criteria for selecting it | Thank you for helping us clarify and update our analysis plan.  The most suitable correlation structure will be determined from the data based on multiple measures, including Akaike’s and Bayesian Information Criteria. This is now included in our updated data analysis plan, please see lines 301-308. |
| 3- Identify the software that will be used for statistical analysis. | Please see lines 309-310. |
| **REV #2** |  |
| The authors should illustrate the central sensitization of the brain and how the online interventions overcome central sensitization in detail | Thank you for this comment and the opportunity to further clarify these entities.  Please see the following additions:  lines 67-71: These predisposing, triggering and maintaining factors lead to structural, functional and chemical changes in the central nervous system causing alterations in processing of internal and external stimuli, i.e. central sensitization. The mechanisms of central sensitization have been widely studied among patients with chronic pain  lines 144-145: Meditation training has been shown to decrease the reactivity of the amygdala to emotional stimuli also during non-meditative states.    lines 150-152: Overall, there is strong evidence of the structural and functional changes in the brain following mindfulness meditation in numerous conditions, including chronic pain, a widely studied condition of central sensitization. |
| The study included 360 participants. Dose participant heterogeneity have an impact on the results. For example, age/gender? | Thank you for the possibility to clarify the manuscript on this topic. Please see additions made on lines 287-288:  We will perform a modifier analysis on baseline characteristics that may influence outcome impact.  The Discussion already states the following on lines 391-393:  Having enough patients and randomizing them to the different study arms aims at reducing the effect of possible baseline confounding factors. |
| The framework of the paper needs to be readjusted. The discussion part is the fifth part, but there are no 1-4 in the manuscript. | Thank you for noticing this error. We have removed ‘5.’ from the ‘Discussion’ section. |
| Additionally, the paper lack results section. | Please see line 332-334:  We have added the following on line 332: ‘The trial is still ongoing.’ |
